# Lipodystrophy defined by Fat Mass Ratio in HIV-infected patients is associated with a high prevalence of glucose disturbances and insulin resistance

**DOI:** 10.1186/1471-2334-12-180

**Published:** 2012-08-06

**Authors:** Paula Freitas, Davide Carvalho, Ana Cristina Santos, Joana Mesquita, Maria João Matos, Antonio Jose Madureira, Esteban Martinez, António Sarmento, José Luís Medina

**Affiliations:** 1 Department of Endocrinology, Hospital de São João, University of Porto Medical School, Alameda Hernâni Monteiro, 4200, Porto, Portugal, Portugal; 2 Department of Endocrinology, Hospital de São João, University of Porto Medical School, Porto, Portugal; 3 Department of Clinical Epidemiology, Predictive Medicine and Public Health, University of Porto Medical School, and University of Porto Institute of Public Health, Porto, Portugal; 4 Department of Endocrinology, Hospital de São João, University of Porto Medical School, Porto, Portugal; 5 Department of Endocrinology, Hospital de São João, University of Porto Medical School, Porto, Portugal; 6 Department of Radiology, Hospital de São João, University of Porto Medical School, Porto, Portugal; 7 Department of Infectious Diseases, Hospital Clinic, University of Barcelona Medical School, Barcelona, Spain; 8 Department of Infectious Disease, Hospital de São João, University of Porto Medical School, Porto, Portugal; 9 Department of Endocrinology, Hospital de São João, University of Porto Medical School, Porto, Portugal

**Keywords:** Lipodystrophy, Insulin resistance, HIV, Glucose homeostasis disturbances

## Abstract

**Introduction:**

Combined antiretroviral therapy (cART) in the treatment of HIV-1 infection has been associated with complications, including lipodystrophy, hyperlipidaemia, insulin resistance (IR) and diabetes.

**Aims:**

To compare the prevalence of glucose homeostasis disturbances and IR in HIV patients on cART according to the presence of lipodystrophy (defined clinically and by Fat Mass Ratio) and different patterns of fat distribution and to establish their associations.

**Design:**

Cross-sectional cohort study.

**Methods:**

We evaluated body composition and IR and insulin sensitivity indexes in 345 HIV-infected adults.

**Results:**

Patients with clinical lipodystrophy (CL) had higher plasma glucose levels than patients without CL, without significant differences in plasma insulin levels, A1c, HOMA-IR, HOMA-B, QUICKI, or MATSUDA index. Patients with lipodystrophy defined by FMR had higher plasma glucose and insulin levels, A1c, HOMA-IR, QUICKI and MATSUDA than patients without lipodystrophy, without differences in HOMA-B. Higher insulin resistance (HOMA-IR ≥ 4) was present in patients with FMR-defined lipodystrophy. Patients with FMR-defined lipodystrophy had a higher prevalence of IFG, IGT and DM than patients without lipodystrophy. Significant associations between HOMA-IR and total, central and central/peripheral fat evaluated by CT at abdominal level were found and no association between HOMA-IR and peripheral fat. Association between HOMA-IR and total and trunk fat but no association with leg and arm fat (evaluated by DXA) was found.

**Conclusions:**

IR and glucose disturbances were significantly increased in patients with FMR-defined lipodystrophy. FMR lipodystrophy definition seems to be a more sensitive determinant of insulin resistance and glucose disturbances than clinical definition.

## Introduction

Combined antiretroviral therapy (cART) in the treatment of HIV-1 infection confers significant survival benefit and has altered the natural history of this disease [1]. However, cART has been associated with metabolic complications, including hyperlipidemia, insulin resistance (IR), diabetes mellitus (DM) and lipodystrophy, with resultant increased risk of cardiovascular disease [2].

DM is a known complication of several antiretroviral therapies, being associated with both short [3,4] and long-term [5,6] exposure to some of these drugs [7]. The reported prevalence of DM in HIV-infected populations ranges from 2 % to 14 %, with differences in prevalence explained by differences in demographic characteristics, lifestyle, and antiretroviral exposure [7-11]. Some studies have suggested an increased risk of premature cardiovascular disease in HIV-infected individuals, and have highlighted the need to understand the relationship of HIV infection and cART with the risk of DM, a primary cardiovascular risk factor [12,13]. Diabetes is associated with IR, and IR among treated HIV-infected patients is multifactorial: in addition to the common contributors to IR (e.g. obesity, physical inactivity and genetic influences), antiretroviral drugs and lipodystrophy or alterations in body fat distribution are also involved [5]. The term “HIV-associated lipodystrophy syndrome” was coined, but it soon became clear that some patients have pure lipoatrophy, while others have central fat accumulation, and a subset of patients have a mixed picture of both morphologic features. As in congenital lipodystrophy, lipodystrophy related to HIV-infected patients is also associated with IR and overt DM [12,14,15].

The aim of this study was to compare the prevalence of glucose homeostasis disturbances and IR in HIV-infected adult patients on cART according the presence of lipodystrophy [clinically defined and FMR-defined determined by whole-body dual-energy X-ray absorptiometry (DXA)] and to different patterns of fat distribution, and to establish the main contributors to these alterations in HIV-infected adults.

## Methods

### Subjects

As part of a cross-sectional cohort study, 345 HIV-infected Caucasian adults, 239 men and 106 women, who were non-institutionalized, were evaluated in the Endocrinology Outpatient Department of São João Hospital, consecutively referred from the Infectious Diseases Clinic. Patients were included on the first visit, and all were on cART. The study protocol was approved by the Hospital Ethics Committee for Health and all patients provided informed consent.

### Clinical assessment

For each patient the following information was collected using a standardized protocol: demographic data (age, gender), duration of HIV infection, HIV infection risk factors, duration of cART and characterization of the infection. We used the “Centers for Disease Control and Prevention” (CDC) criteria for classifying the degree of infection [16]. Clinical history of diabetes, hypertension and use of anti-diabetic, anti-hypertensive and lipid-lowering drugs, as well as duration of cART, were also evaluated. Weight, height, circumferences of neck, waist, hip, thigh and arm were measured as previously published [17-20]. Blood pressure (BP) was measured after 5 minutes seated, with the elbow flexed at the heart, using a standard aneroid sphygmomanometer with the cuff on the upper right arm. Two blood pressure readings were taken and the mean of the two readings was calculated. Body mass index (BMI) was calculated as weight divided by height squared (kg/m^2^). Clinical lipodystrophy was defined as a peripheral lipoatrophy with or without central fat accumulation assessed by both patient and practitioner, as we have previously described [17-20]. Presence of central fat accumulation or abdominal prominence was defined by the measurement of waist circumference using the International Diabetes Federation (IDF) criteria for metabolic syndrome [21].

Patients were classified into 4 different groups according the presence or absence of either clinical lipoatrophy or abdominal prominence: **no lipodystrophy** - patients without clinical lipoatrophy and without abdominal prominence; **isolated central fat accumulation** - patients without clinical lipoatrophy and with abdominal prominence; **isolated lipoatrophy** - patients with clinical lipoatrophy and without abdominal prominence; **mixed forms of lipodystrophy** - patients with clinical lipoatrophy and with abdominal prominence. The clinical assessment was performed by the same practitioner (PF).

### Evaluation of body composition

Body composition was also assessed with whole-body dual–energy X-ray absorptiometry (DXA – Lunar Expert XL). DXA measurement was performed while the patient was in a supine position, with standard positioning of the arms and feet. Markers used in this study for trunk and lower limbs that defined regions of interest were those indicated by the manufacturer. Regional fat mass values were grouped and analysed for the following anatomical regions: arms, legs, trunk and total body. The fat mass ratio (FMR) is the ratio of the percentage of the trunk fat mass to the percentage of the lower limb fat mass (FMR = % of the trunk fat mass/ % of the lower limb fat mass) [22]. We define lipodystrophy by FMR using the cut-off value of 1.961 threshold for men and 1.329 for women [17].The quantification of total, visceral, and peripheral fat was performed with a 64-slice computed tomography (CT) scanner (Siemens Sensation 64 Cardiac) with the same technique as previously described [23,24]. All values were expressed in cm^2^ rounded to the nearest centesimal.

### Laboratory analysis

A venous blood sample was drawn after a 12-hour overnight fast. All the samples were analysed at the central laboratory of our hospital. Patients without a previous diagnosis of diabetes were submitted to a glucose tolerance test (OGTT). The test was performed as described by the World Health Organization using a glucose load containing the equivalent of 75 g anhydrous glucose dissolved in water.

Hepatitis C was diagnosed by HCV-Ab serostatus. The CD4 cell count was determined by flow cytometry and plasma HIV-1 RNA loads were measured by a quantitative reverse transcriptase polymerase chain reaction (Roche Diagnostic Systems, Inc., Branchburg, NJ, USA), which has a lower limit of detection of 50 copies/mL.

### Definition of the alterations of glucose metabolism

Diabetes was defined if two consecutive measurements of fasting plasma glucose were ≥126 mg/dL and if 2 h plasma glucose was ≥200 mg/dL during the oral glucose tolerance test (OGTT).

Alterations of glucose metabolism were defined by American Diabetes Association (ADA) 2009 criteria [25]. We classified patients into 4 different categories of glycaemic profile (normoglycaemia – NG, impaired fasting glucose – IFG, impaired glucose tolerance – IGT and diabetes mellitus type 2 – DM), according to plasma glucose levels at 0’ and 120’ measured during OGTT.

### Measurements of insulin resistance

IR was defined by the homeostasis model assessment of insulin resistance (HOMA) and insulin sensitivity by the quantitative insulin sensitivity check index (QUICKI) and Matsuda index. These indexes were calculated using the following formulas:$HOMA-IR\;index=\left(\text{fasting insulin }0\text{ x fasting glucose }0\right)/22.5$[26].$HOMA\;B\;index=\left(20\text{x insulin }0\;\left(\text{mU}/\text{mL}\right)\right)/\left(\text{glucose }0\;\left(\text{mMol}/\text{L}\right)-3.5\right)$[26].$QUICKI=1/(\text{log}\left[\text{fasting insulin in mU}/\text{l}\right]+\text{log}\left[\text{fasting plasma glucose in mg}/\text{dL}\right]$[27].$Matsuda\;Inde\text{x }=10\;000/\text{sqrt}\left[\text{glyc }0\text{x insulin }0\text{x}\left(\text{glyc mean x insulin mean}\right)\right]$[28].Glucose expressed in mmol/L and insulin inmUI/mL. IR was defined when the value of HOMA >4[29,30].

### Statistical analysis

Statistical analysis was performed using SPSS version 17.0 software (SPSS Inc., Chicago, Illinois, USA). All probabilities were two tailed and p values <0.05 were regarded as significant.

Data were described as mean and standard deviation (SD) for quantitative variables and compared using the Student-t test or the Mann–Whitney test as appropriate. Furthermore, analysis of variance or the Kruskal-Wallis test were used for the comparison between quantitative variables. Categorical variables were described as counts and proportions, and compared using the chi-square or Fisher’s exact test.

Spearman correlation coefficients were calculated to estimate the associations between HOMA-IR and body fat mass evaluated either by CT or DXA.

After log transformation, linear regression models were computed for estimating the association between FMR and HOMA score, fasting and 2 hours glucose and A1C levels. Models were adjusted for age, sex, current antiretroviral therapy [protease inhibitor (PI), nucleoside reverse transcriptase inhibitor (NRTI), non-nucleoside reverse transcriptase inhibitor (NNRTI)], CD4 count cells and plasma HIV-1 RNA load.

## Results

### Baseline characteristics

A total of 345 HIV-infected patients on cART were evaluated with a mean age of 45.0 ± 11.3 years. 69.3 % were males. The demographic and clinical characteristics of the patients included in this study, according to the presence of lipodystrophy are shown in Table 1. Patients with clinical lipodystrophy (CL) were older, had higher duration of HIV infection and length of cART. Regarding anthropometric measures, patients with CL had lower BMI, waist, hip, thigh, and arm circumferences. Patients with CL had higher CD4+ cell count and percentage of viral suppression. With regards to the risk of HIV transmission in patients with CL, heterosexuality was responsible for 52.9 %, followed by drug addiction in 30.6 %, homo-bisexuality in 11.2 %. In the remaining cases, haemophilia, transfusions and unknown causes were responsible for 1.2 %, 1.2 % and 2.9 %, respectively. Similarly, in patients without CL, heterosexuality was also the leading cause with 65.8 %, followed by drug addiction, homo-bisexuality and unknown causes in 24.6 %, 8.8 % and 0.9 %, respectively. No significant difference was observed in lipodystrophic patients according to HIV risk transmission. The prevalence of co-infection was similar in patients with and without lipodystrophy [clinically- (Table 1) or FMR-defined (data not shown)]. Nevertheless, patients with no lipodystrophy and with isolated peripheral lipoatrophy exhibited a higher proportion of hepatitis C co-infection when compared to the other two groups (Table 2).

**Table 1:** Sample characteristics according to the presence of clinical lipodystrophy

	**Without CL**	**With CL**	***P***
n (%)	139	206	
Gender [n (%)]			
Male	85 (61.2)	154 (74.8)	0.007
Female	54 (38.8)	52 (25.2)	
Age [years, mean (sd)]	44.2 (12.1)	46.9 (10.7)	0.007
Duration of HIV infection [years, median (IQR)]	6.0 (5.0)	9.0 (5.0)	<0.001
cART [years, median (IQR)]	4.0 (5.0)	8.0 (5.0)	<0.001
Weight [Kg, mean (sd)]	73.7 (14.1)	66.2 (12.4)	<0.001
Height [m, mean (sd)]	1.65 (0.09)	1.66 (0.09)	0.368
BMI [(kg/m^2^), median (IQR)]	26.3 (5.9)	23.8 (5.1)	<0.001
Waist circumference [cm, median (IQR)]	94.0 (17.0)	89.0 (14.5)	<0.001
Hip circumference [cm, median (IQR)]	99.0 (11.0)	91.0 (9.0)	<0.001
Thigh circumference [cm, mean (sd)]	50.0 (5.5)	46.1 (6.1)	<0.001
Arm circumference [cm, mean (sd)]	28.1 (3.1)	26.6 (4.1)	<0.001
Neck circumference [cm, mean (sd)]	37.2 (3.7)	37.1 (4.0)	0.940
CD4 cell count [cells/mm3, median (IQR)]	446.5 (342.2)	544.0 (387.0)	0.008
HIV RNA (<50) [n (%)]	93 (81.6)	163 (91.1)	0.017
Smoking history [n (%)]			
Never	61 (44.2)	70 (34.8)	0.219
Current	55 (39.9)	93 (46.3)	
Former	22 (15.9)	38 (18.9)	
HIV risk factor [n (%)]			
Injecting drug user	28 (24.6)	52 (30.6)	0.247
Homosexual contact	10 (8.8)	19 (11.2)	
Heterosexual contact	76 (65.8)	90 (52.9)	
Others	1 (0.9)	9 (5.3)	
Hepatitis C co-infection [n (%)]	31 (25.2)	58 (31.4)	0.300
CDC [n (%)]			
A	62 (54.9)	94 (53.4)	0.037
B	4 (3.5)	0 (0)	
C	47 (41.6)	82 (46.6)	
ART [n (%)]			
IP	67 (61.5)	98 (55.4)	0.310
NNRTI	47 (43.1)	83 (46.9)	0.534
NRTI	104 (95.4)	175 (98.9)	0.110
Hypoglycemic therapy [n (%)]			
Oral anti-diabetic drugs	10.1 % (14)	14.6 % (30)	0.132
Insulin	4.3 % (6)	7.3 % (15)	0.261
Body fat mass by quantitative CT			
Total fat [cm^2^, median (IQR)]	340.5 (196.6)	213.6 (175.2)	<0.001
Central fat [cm^2^, median (IQR)]	110.8 (111.4)	114.8 (120.5)	0.800
Peripheral fat [cm^2^, median (IQR)]	186.2 (172.0)	78.7 (104.9)	<0.001
Central/peripheral fat ratio [cm^2^, median (IQR)]	0.51 (0.55)	1.34 (2.15)	<0.001
FMR by DXA [median (IQR)]	1.06 (0.57)	1.79 (1.37)	<0.001

(CL- clinical lipodystrophy; CDC - Centers for Disease Control and Prevention criteria for staging of HIV infection -; cART- combined antiretroviral therapy; BMI- body mass index; FMR – fat mass ratio; DXA –dual-energy X-ray absorptiometry ; CT – computed tomography; PI – protease inhibitor; NNRTI –non-nucleoside reverse transcriptase inhibitor; NRTI – nucleoside reverse transcriptase inhibitor).

**Table 2:** Smoking history and hepatitis C co-infection according to the four groups of body fat distribution

	**No lipodystrophy**	**Isolated central fat accumulation**	**Isolated peripheral lipoatrophy**	**Mixed forms of lipodystrophy**	**P**
Smoking history [n (%)]					
Never	14 (28.0)	46 (52.9)	24 (22.4)	45 (48.4)	<0.001
Current	30 (60.0)	25 (28.7)	67 (62.6)	26 (28.0)	
Former	6 (12.0)	16 (18.4)	16 (15.0)	22 (23.7)	
Hepatitis C co-infection [n (%)]	19 (45.2)	12 (14.8)	46 (47.9)	11 (12.5)	<0.001

There was a significant difference in the different stages of CDC criteria between patients with or without lipodystrophy, with the majority of patients being in groups A or C. No differences were observed in the type of cART between the two groups of patients, nor in hypoglycaemic therapy (oral anti-diabetic drug and insulin). FMR evaluated by DXA was higher in patients with CL. When body fat mass was evaluated using quantitative CT, patients with CL had lower total and peripheral fat, but higher central/peripheral fat ratio than patients without CL.

No differences in smoking status between patients with or without lipodystrophy [clinically- (Table 1) or FMR-defined (data not shown)] were found. Patients with no lipodystrophy and with isolated peripheral lipoatrophy were more frequently current smokers when compared to the other two groups (Table 2).

### Insulin resistance

No significant differences in the means of HOMA-IR, QUICKI, MATSUDA, insulin and A1c were observed between patients with and without CL. In fact, regarding the alterations of glucose metabolism, only for fasting glucose was there a trend for significantly higher values in CL. On the other hand, when lipodystrophy was defined by FMR, all indicators of insulin resistance and glucose metabolism were significantly associated with lipodystrophy with the obvious exception of QUICKI and MATSUDA indices (Table 3).

**Table 3:** Insulin resistance indices according to lipodystrophy defined clinically and by FMR

**Lipodystrophy defined clinically**					**defined by FMR**		
	**Total**	**Without CL**	**With CL**	**P**	**Without L**	**With L**	**P**
HOMA-IR [median (IQR)]	1.9 (2.8)	1.7 (2.4)	2.1 (3.0)	0.229	1.6 (2.6)	2.8 (2.8)	<0.001
HOMA-B [median (IQR)]	92.3 (106.4)	92.3 (91.1)	91.8 (117.5)	0.855	92.3 (96.1)	85.4 (120.8)	0.633
QUICKI [median (IQR)]	0.34 (0.06)	0.35 (0.06)	0.34 (0.06)	0.229	0.36 (0.07)	0.33 (0.05)	<0.001
MATSUDA [median (IQR)]	4.8 (5.1)	5.0 (4.2)	4.7 (5.4)	0.735	5.9 (5.3)	4.1 (4.4)	0.002
Glucose [mg/dL, median (IQR)]	93.5 (26.0)	90.0 (23.0)	97.0 (35.5)	0.046	90.0 (23.0)	97.0 (35.5)	0.002
Insulin [μU/mL, median (IQR)]	8.1 (9.6)	7.9 (8.2)	9.0 (10.2)	0.365	7.5 (8.1)	10.1 (9.9)	0.003
A1c [% median (IQR)]	5.3 (0.7)	5.2 (0.6)	5.3 (0.9)	0.091	5.2 (0.6)	5.5 (0.9)	0.002

(CL- clinical lipodystrophy; L- lipodystrophy).

Higher prevalence of insulin resistance, defined as HOMA-IR ≥ 4, was observed in patients with lipodystrophy defined by FMR (p = 0.019) but not when lipodystrophy was clinically defined. Similar results were observed when we compared the prevalence of HOMA score thirds according to the definition of lipodystrophy. Again, only when lipodystrophy was defined by the FMR were the differences between HOMA score thirds statistically significant (p = 0.002) (Table 4).

**Table 4:** Insulin resistance according to lipodystrophy defined clinically and by FMR

	**Lipodystrophy defined clinically**			**Lipodystrophy defined by FMR**		
	**Without CL**	**With CL**	**P**	**Without L**	**With L**	**P**
HOMA –IR						
< 4 [n (%)]	93 (80.9)	121 (71.6)	0.075	94 (80.3)	58 (65.9)	0.019
≥ 4 [n (%)]	22 (19.1)	48 (28.4)		23 (19.7)	30 (34.1)	
Thirds of HOMA-IR						
1	40 (34.8)	55 (32.5)	0.222	51 (43.6)	18 (20.5)	0.002
2	43 (37.4)	51 (30.2)		34 (29.1)	33 (37.5)	
3	32 (27.8)	63 (37.3)		32 (27.4)	37 (42.0)	

(CL- clinical lipodystrophy; L- lipodystrophy).

### Glucose homeostasis abnormalities

When we classified patients into the 4 ADA categories of glycaemic profile, no significant differences were found between these categories in patients with or without CL. However, patients with lipodystrophy defined by FMR had a higher prevalence of IFG, IGT and DM when compared to patients without lipodystrophy (Table 5). When patients were stratified into 4 groups of fat distribution (presence or not of clinical lipoatrophy and abdominal prominence), no differences were observed in glycaemic profile. However, when we divided patients according to the 4 categories of fat distribution (presence or not of lipodystrophy defined by FMR and abdominal prominence), patients with abdominal prominence independent of the presence of lipodystrophy had higher IGT. Moreover, the highest prevalence of DM was observed in patients with lipodystrophy and abdominal prominence (Table 6).

**Table 5:** Prevalence of glucose homeostasis abnormalities according to lipodystrophy defined clinically and by FMR

	**Lipodystrophy defined clinically**				**Lipodystrophy defined by FMR**		
	**Total (n = 228)**	**Without CL (n = 89)**	**With CL (n = 129)**	**P**	**Without L (n = 100)**	**With L (n = 70)**	**P**
NG [n (%)]	117 (49.1)	44 (49.4)	63 (48.8)	0.364	62 (62.0)	29 (41.4)	0.021
IFG [n (%)]	18 (8.3)	10 (11.2)	8 (6.2)		5 (5.0)	11 (15.7)	
IGT [n (%)]	49 (22.5)	21 (23.6)	28 (21.7)		19 (19.0)	15 (21.4)	
DM [n (%)]	44 (20.2)	14 (15.7)	30 (23.3)		14 (14.0)	15 (21.4)	

(NG- normal glucose; IFG – impaired fasting glucose: IGT – impaired glucose tolerance; DM – diabetes mellitus; CL – clinical lipodystrophy; L – lipodystrophy; L-lipodystrophy).

**Table 6:** Prevalence of glucose homeostasis abnormalities according to body composition categorised into 4 groups of fat distribution

**Categories of fat distribution by clinical lipoatrophy and WC**						**Categories of fat distribution by FMR and WC**				
	**CLA- AP-**	**CLA-AP+**	**CLA + AP-**	**CLA + AP+**	**P**	**L- AP-**	**L-AP+**	**L + AP-**	**L + AP+**	**P**
NG [n (%)]	17 (56.7)	27 (45.8)	41 (58.6)	22 (37.3)	0.064	39 (61.9)	32 (50.8)	19 (51.4)	17 (30.9)	0.002
IFG [n (%)]	1 (3.3)	9 (15.3)	4 (5.7)	4 (6.8)		0 (0.0)	7 (11.1)	5 (13.5)	6 (10.9)	
IGT [n (%)]	5 (16.7)	16 (27.1)	10 (14.3)	18 (30.5)		9 (14.3)	17 (27.0)	6 (16.2)	17 (30.9)	
DM [n (%)]	7 (23.3)	7 (11.9)	15 (21.4)	15 (25.4)		15 (23.8)	7 (11.1)	7 (18.9)	15 (27.3)	

(NG- normal glucose; IFG – impaired fasting glucose: IGT – impaired glucose tolerance; DM – diabetes mellitus; CLA – Clinical lipoatrophy; AP – abdominal prominence defined by waist circumference >80 cm in women and >94 cm in men – IDF 2005; L- lipodystrophy FMR defined; CLA-AP-: no lipodystrophy; CLA-AP+: isolated central fat accumulation; CLA + AP-: isolated lipoatrophy; CLA + AP+: mixed forms of lipodystrophy).

### Association between HOMA-IR and body fat mass evaluated by CT and DXA

After log transformation, significant associations were found between total fat, central fat and central/peripheral fat ratio evaluated by CT at abdominal level and HOMA-IR. These associations remained significant after adjustments for age, gender, PI, NRTI, NNRTI, CD4 cell count and HIV-1 RNA viral load. On the other hand, no significant association was found between IR and peripheral fat. Similar results were observed when fat distribution was evaluated by DXA. Significant associations were found between total, trunk and arm fat mass evaluated by DXA and HOMA-IR. Also, these associations remained significant after adjustments for age, gender, PI, NRTI, NNRTI, CD4 cell count and HIV-1 RNA viral load (Table 7).

**Table 7:** Association between HOMA-IR and body fat mass evaluated by CT and DXA

	**Crude model**		Model 1*		Model 2**	
	**β**	**P**	**β**	**P**	**β**	**p**
Body Fat Mass by Quantitative CT						
Total Fat	0.384	0.001	0.419	0.001	0.415	0.001
Central Fat	0.516	<0.001	0.486	<0.001	0.490	<0.001
Peripheral Fat	0.050	0.530	0.127	0.168	0.114	0.222
Central/peripheral fat ratio	0.258	0.001	0.239	0.015	0.262	0.008
Body fat mass by DXA						
Total	0.247	0.024	0.374	0.002	0.420	0.001
Trunk	0.403	<0.001	0.467	<0.001	0.502	<0.001
Leg	−0.043	0.625	0.049	0.653	0.087	0.438
Arm	0.162	0.058	0.352	0.001	0.366	0.001

*-Mode1: adjusted for age and gender.

**-Model 2: adjusted for age, gender, IP, NRTI, CD4 cell count and HIV-1 RNA viral load.

(DXA –dual-energy X-ray absorptiometry; CT – computed tomography).

### Association between FMR and glycaemic parameters and insulin resistance

After log transformation, a significant positive linear association was observed between FMR and insulin resistance evaluated by HOMA. This association was independent of age, gender, IP, NRTI, NNRTI, CD4 cell count and HIV-1 RNA viral load. No significant association was observed with the glycaemic parameters (Table 8). We performed linear regression models of the association between FMR and the variables described above, after excluding patients that were on anti-diabetic or insulin therapy, and similar results were obtained (data not shown).

**Table 8:** Association between FMR and glycaemic parameters and insulin resistance

	**Crude model**		Model 1*		Model 2**	
	**β**	**P**	**β**	**P**	**β**	**p**
HOMA	0.589	<0.001	0.595	0.001	0.266	0.002
Glucose 0´	0.124	0.002	0.041	0.367	0.098	0.222
Glucose 2 h	0.079	0.142	0.086	0.139	0.149	0.104
A1C	0.053	0.015	0.007	0.765	0.083	0.314

*-Mode1: adjusted for age and gender.

**-Model 2: adjusted for age, gender, IP, NRTI, CD4 cell count and HIV-1 RNA viral load.

## Discussion

We compared the prevalence of glucose homeostasis disturbances and IR in HIV-infected patients on cART, according to the presence of lipodystrophy (clinically and FMR defined) and according to the four different patterns of body fat distribution previously described. In our study, all the participants were evaluated by the same observer (PF) to increase the accuracy in the clinical definition of lipodystrophy [22]. We also used an objective method to evaluate lipodystrophy to overcome the different and heterogeneous methodologies for the diagnosis of lipodystrophy of previous studies [31]. Since lipodystrophy in HIV-1 infected patients is considered to be an adverse effect of cART, not limited to a specific drug or a class of drugs [32], we chose to study only patients on cART, since our main goal was to compare the prevalence of glucose homeostasis disturbances and IR according to the presence of lipodystrophy. No differences were observed between the class of drugs used in patients with or without CL, defined either clinically or by FMR (data not shown). Also, similar results were observed when the comparison was performed in patients with and without lipodystrophy defined by FMR (data not shown). Despite the euglycaemic-hyperinsulinaemic clamp being the gold standard technique for estimation of IR [33], we used simple methods that have shown a good correlation with the gold standard method (HOMA-IR, QUICKI and MATSUDA) [28,34]. There are studies comparing the prevalence of DM in HIV patients and the general population, and comparing ART-naïve HIV-infected patients with the general population, but fewer compared this prevalence between patients with or without lipodystrophy.

When patients were classified as being lipodystrophic or not, according to FMR, we observed that patients with lipodystrophy had higher IR (higher HOMA and lower QUICKI and Matsuda values). Matsuda index seems to have a greater ability to predict diabetes than its HOMA equivalents [34]. They also had higher fasting plasma glucose, insulin and A1C levels, and higher % of IFG, IGT and DM. When we categorised patients into 4 categories of body fat distribution using FMR-defined lipodystrophy and waist circumference, those patients with lipodystrophy and abdominal prominence had higher prevalence of DM and IGT. Patients without FMR-defined lipodystrophy but with abdominal prominence only had a high prevalence of IGT. It seems that the loss of peripheral adipose tissue is less important than the presence of abdominal prominence in the occurrence of IR. However, the role of peripheral adipose tissue cannot be completely precluded, since patients with abdominal prominence only and without lipodystrophy, defined by FMR, had less marked glucose disturbances i.e. they only had increased prevalence of IGT. The discrepancy observed between the results obtained using the 2 different lipodystrophy definitions (Tables 3, 5 and 6) could result from the higher accuracy of the objective method in detecting slight losses of peripheral adipose tissue that were not detected by clinical inspection, as has been previously proposed by Bonnet [22].

Significant associations between IR and total fat, central fat and central/peripheral fat ratio and no association with peripheral fat at abdominal level evaluated by CT were observed, emphasizing the contribution of the central fat mass to IR. We found an association between IR and total and trunk fat evaluated by DXA. As in our results, De Wit et al. showed that clinical lipodystrophy was significantly associated with new-onset diabetes and the abnormal body fat distribution in HIV-positive individuals is strongly associated with IR and/or glucose intolerance, with excess trunk or visceral fat being, as in the general population, an important risk factor for IR among those with HIV infection. In addition, De Wit observed that IR is itself independently associated with fat loss in HIV-positive individuals [5].

In our sample, no association was observed between peripheral fat mass and IR (Table 7). This result is contrary to that of the De Wit study, where patients had a marked peripheral adipose tissue loss that was clinically evident. Also, our patients may have had less evident loss of fat mass, which was detected by FMR. In fact, according to De Wit’s conclusions, information on lipodystrophy was rarely collected since the study was carried out in 11 centres, which may suggest that only marked lipodystrophies were reported.

The dual contribution of peripheral fat loss and increased abdominal fat has also been described by other authors [35-38]. Furthermore, hyperinsulinaemia seems to be more severe among the patients with a combined fat redistribution syndrome.

In our HIV-infected sample, we had a high prevalence of diabetes (20.2 %), of IGT (22.5 %) and IFG (8.3 %). This high prevalence could not be extrapolated for the total HIV population since we could have a referral bias, but we would like to emphasize that our aim was to evaluate the prevalence of glucose homeostasis disturbances and IR according to the presence of lipodystrophy. In HIV-infected patients, some studies showed a low prevalence of diabetes, around 3.0 %, but without performing OGTT, which could have resulted in some under-identification of glucose disturbances [39]. Others suggested that the prevalence and incidence of diabetes was higher (10-14 %) [7,8,40-42] but still lower than that which we observed. Concerning HIV-associated lipodystrophy, Carr found a baseline diabetes prevalence of 2 % [43] and an over threefold increase after 14 months of follow-up [44], which suggests that lipodystrophy strongly promotes the progression to hyperglycaemia. The overall prevalence of all glucose disturbances (DM, IFG and IGT) was 25 % [43]. In the Lipodystrophy Case Definition Study, diabetes prevalence was 7 % in those with lipodystrophy and 3 % in those without [45].

It is extremely difficult to determine which drug is responsible for the risk of diabetes because they are always used in combination and therapeutic changes in individual participants often occur during the course of the disease. The results are generally consistent, indicating a higher risk of diabetes with use of NRTI and NNRTI [46,47]. Also, others have shown that regimens containing PI are associated with new-onset diabetes and IR [48,49]. cART has direct effects on glucose metabolism, or exerts its effect on glucose metabolism indirectly by affecting changes in body composition. PIs directly and/or indirectly alter body composition, lipid profile, adipokine levels, and mediators of inflammation. The newer cART regimens may be less detrimental to insulin action than older regimens.

In our study, FMR as a continuum was significantly associated with insulin resistance evaluated by HOMA, even after adjustment for gender, age, IP, NRTI, NNRTI, CD4 cell count and HIV RNA viral load. No association between clinical and FMR-defined lipodystrophy and glycaemic parameters was observed.

The association between fat redistribution, glucose disturbances, IR and cART, might be explained through several mechanisms: lipoatrophy per se; lipotoxicity (cART or visceral fat-induced or both, demonstrated by increased lipolysis and circulating free fatty acids) [2]; inflammation (resulting from the infection and demonstrated by increased TNF-alpha production, increased serum concentrations of the soluble type 2 receptor of TNF-alpha [39] and other interleukins), and hormonal factors (leptin, resistin or adiponectin).

We should keep in mind that IR and diabetes are associated with age, so increased life expectancy with the use of cART is likely to increase the prevalence of diabetes [7].

The net risk of diabetes mellitus is determined by a complex interplay of individual factors, combining the traditional risk factors and newer risk factors associated with HIV-infected patients on cART. The clinical implications and message should be the need to aggressively screen for, prevent, and treat diabetes mellitus among HIV-infected patients because the presence of diabetes increases the risk of a future CHD event by almost 2.5 times [7].

### Our study has some limitations

Although all patients referred from Infectious Diseases to our department were included, bias in the referral cannot be excluded, since some patients could have been referred because they had some degree of glucose intolerance or dyslipidaemia. It was not our aim to determine the prevalence of glucose homeostasis disturbances in the HIV population, because these patients are not representative of the general HIV population in our country. Our aim was to compare the prevalence of glucose homeostasis disturbances and IR in HIV-infected patients according the presence of lipodystrophy and to different patterns of fat distribution.

Also, information regarding other important risk factors for DM (e.g. family history, physical inactivity) was not collected and could not be analysed. Some drugs used for managing complications of HIV infection may be associated with worsening IR or DM; we believe that if there was any effect it was very small, since none of our patients were on niacin, megestrol acetate or steroids, and only some of them were on thiazides for the management of hypertension.

### Some aspects of this study should be highlighted

The study was performed in a unit that is highly experienced in the assessment of metabolic and body fat abnormalities in HIV-infected patients; the clinical assessment of lipodystrophy was carried out by the same investigator (PF); and an objective definition of lipodystrophy (Fat Mass Ratio by DXA), visceral and subcutaneous fat mass by CT was used.

## Conclusions

Insulin resistance and glucose homeostasis abnormalities significantly increased with increasing FMR. Abdominal prominence, measured by abdominal circumference, waist/hip ratio, visceral and trunk fat defined by CT, was also associated with glucose homeostasis disturbances.

Although no association between glucose disturbances and IR was observed in clinically defined lipodystrophy, when this condition was defined by FMR, a significant positive association was observed.

## Competing interest

None to declare.

## Authors’ contributions

PF conceived the study, participated in its design, in the acquisition of data and drafted the manuscript; DC conceived the study, participated in its design and drafted the manuscript; ACS performed the statistical analysis and revised critically the manuscript; JM participated in the acquisition of data and drafted the manuscript; MJM participated in the acquisition of data; AJM performed the CT scan and reviewed the data; EM and AS critically revised the manuscript; JLM revised the study design. All authors read and approved the final manuscript.

## Funding

Research Fellowship Dr. Manuel Almeida Ruas, Portuguese Society of Diabetology. Research Fellowship of the Portuguese Association for Clinical Study of AIDS. Research Grant to support doctoral studies in the area of HIV/AIDS, GlaxoSmithKline Foundation of Health Sciences.

## Pre-publication history

The pre-publication history for this paper can be accessed here:

http://www.biomedcentral.com/1471-2334/12/180/prepub
